# First evaluation of a symbiotic food supplement in an allergen exposure chamber in birch pollen allergic patients

**DOI:** 10.1016/j.waojou.2020.100494

**Published:** 2020-12-18

**Authors:** Karl-Christian Bergmann, Linda Krause, Julia Hiller, Sylvia Becker, Sebastian Kugler, Martin Tapparo, Oliver Pfaar, Torsten Zuberbier, Matthias F. Kramer, Sonja Guethoff, Anke Graessel

**Affiliations:** a Department for Dermatology and Allergy, Charité *–* Universitätsmedizin Berlin, Corporate Member of Freie Universität Berlin, Humboldt-Universität zu Berlin, And Berlin Institute of Health, Berlin, Germany; b ECARF - European Centre for Allergy Research Foundation, Berlin, Germany; c Institute of Medical Biometry and Epidemiology, University Medical Center Hamburg- Eppendorf, Hamburg, Germany; d Bencard Allergie GmbH, Munich, Germany; e Department of Otorhinolaryngology, Head and Neck Surgery, Section of Rhinology and Allergy, University Hospital Marburg, Philipps-Universität Marburg, Marburg, Germany; f Allergy Therapeutics (UK) Ltd, Worthing, UK

**Keywords:** Symbiotic, Probiotic, Food supplement, Allergen exposure chamber (AEC), Birch pollen allergy, Peak expiratory flow (PEFBackspace), Peak nasal inspiratory flow (PNIF), Total symptom score (TSS)

## Abstract

**Background:**

Allergic rhinitis/rhinoconjunctivitis is the most common immune disease worldwide, but still largely underestimated, underdiagnosed, and undertreated. Dysbiosis and reduced microbial diversity is linked to the development of allergies, and the immunomodulatory effects of pro- and prebiotics might be used to counteract microbiome dysbiosis in allergy. Adequate symbiotic (multi-strain pro-, plus prebiotic) supplementation can be suggested as a complementary approach in the management of allergic rhinitis.

**Objective:**

The effects of the daily intake of a symbiotic food supplement (combination of *Lactobacillus acidophilus NCFM* and *Bifidobacterium lactis BL-04* with Fructo-Oligosaccharides) for 4 months in birch pollen allergic rhinoconjunctivitis patients were investigated for the first time in an allergen exposure chamber (AEC) allowing standardised, reproducible pollen exposure before and after intake.

**Methods:**

Eligible patients were exposed to birch pollen (8000 pollen/m³ for 120 min) at the GA^2^LEN AEC, at baseline (V1) and final visit (V3) outside the season. The Total Symptom Score (TSS) and the scores for nose, eye, bronchial system, and others were evaluated every 10 min during exposure. Other secondary endpoints were the changes in well-being, Peak Nasal Inspiratory Flow (PNIF), lung function parameters, and safety. Co-primary endpoints were differences in Total Nasal Symptom Score (TNSS) and TSS after 120 min of exposure between both visits. Temporal evolution of symptom scores were analysed in an exploratory way using linear mixed effects models.

**Results:**

27 patients (mean age 45 years, 15% male) completed the study. Both co-primary endpoints showed significant improvement after intake of the symbiotic. Median TNSS and TSS were decreased 50% and 80% at 120 min (adjusted p-value = 0.025 and p < 0.01 respectively).

All four symptom scores and the personal well-being, improved to a clinically relevant extent over time, visible by a weaker increase in symptoms during 120 min of the final birch pollen exposure. No relevant differences were observed for PNIF, PEF, and spirometry. There were no airway obstructions or lung restrictions before and after both exposures. Late phase reactions after exposure were reduced after V3, documenting a better birch pollen tolerability of the patients. The safety and tolerability profile of the symbiotic food supplement was excellent, no adverse events (AEs) were observed.

**Conclusions:**

This first evaluation of a symbiotic food supplement in an AEC in rhinoconjunctivitis patients with or without asthma induced by birch pollen revealed a significant beneficial effect, harnessing significant improvements of symptoms and well-being while maintaining an excellent safety and tolerability profile.

## Introduction

The prevalence of allergic diseases is steadily increasing.^1^ Allergic rhinitis (AR)/rhinoconjunctivitis (ARC) is the most common immune disease and one of the most common chronic diseases worldwide.^1^ The socio-economic impact of AR and its comorbidities are considerable for healthcare systems and patients all around the globe.^1^ Almost 1 in 3 Europeans is affected by AR, but it still remains largely underestimated, underdiagnosed, and undertreated.^1^

Allergen immunotherapy (AIT) is well established as the only causative treatment to date,^1^ but there are additional strategies to relieve symptoms of allergic patients, such as the modification of the patient's microbiome and the increase of microbial stimuli from beneficial bacterial strains. Recently, a high number of articles have been published in the field of microbiome research, investigating the effects of the loss of microbial diversity linked to the development of allergies, as described in the hygiene hypothesis and farm effect^2^, ^3^, ^4^, ^5^, ^6^, ^7^, ^8^ Gut microbiota dysbiosis has a high influence on asthma pathogenesis. Dysbiosis and reduced microbial diversity can dysregulate the bidirectional crosstalk along the gut-lung axis, probably resulting in hypersensitivity and hyper reactivity to respiratory as well as food allergens,^9^ also recognized in the field of non-IgE mediated food allergy.^10^

According to the World Health Organization (WHO), probiotics are defined as live microorganisms which need to be administered in adequate amounts to confer a health benefit to the host.^9^^,^^11^^,^^12^ The beneficial properties of probiotics, prebiotics (non-digestible food ingredients selectively stimulating the favourable growth and/or activity of probiotics),^13^ and symbiotics (the combination of probiotics and prebiotics)^13^ only start to be recognized in allergic diseases and allergy prevention.^14^, ^15^, ^16^ Therefore, counteracting microbiome dysbiosis in allergy and asthma using probiotics seems reasonable,^17^^,^^18^ and certain probiotic strains have immunomodulatory effects in favour of a suppression of Th2 and stimulation of a Th1 profile.^13^

The concept of using pro-, pre- and symbiotics in food allergic subjects is currently on the rise in the field of milk allergy^19^ and also analysed in paediatric populations.^20^ Whereas in peanut allergy the concept of oral immunotherapy (OIT) combined with probiotics is under investigation,^21^ in the field of allergies to airborne allergens it has been studied several times before. These studies suggest that certain strains of probiotic bacteria, such as Lactobacilli and Bifidobacteria, can improve allergy symptoms like rhinitis or rhinoconjunctivitis.^22^, ^23^, ^24^, ^25^, ^26^, ^27^, ^28^, ^29^, ^30^, ^31^ Although these studies vary in quality, the effects have shown to be reproducible, and using pre-, pro-, or symbiotics as complementary treatment options in AR seems to be a promising concept.^13^

Pollagen (Germany and Austria, Bencard Allergie GmbH, and Italy, Allergy Therapeutics Italia)/Polagen (Spain, Allergy Therapeutics Ibérica), a symbiotic combination of *Lactobacillus acidophilus NCFM*/*Bifidobacterium lactis BL-04*/Fructo-Oligosaccharides, consumed by patients with clinically documented AR over a period of 4 months reduced the total nasal symptoms and ARIA (Allergic Rhinitis and its Impact on Asthma) classification of rhinitis. This study supports the idea that an adequate symbiotic multi-strain supplementation can be seen as a novel adjuvant concept in the management of seasonal and perennial allergic rhinitis (SAR, PAR). *Lactobacillus acidophilus NCFM* and *Bifidobacterium lactis BL-04*, are relevant candidates for this purpose.^32^ This combination of probiotic strains also prevented the pollen-induced infiltration of eosinophils into the nasal mucosa. This is an objective marker of AR directly correlating with the intensity of the disease, and it indicated a trend for reduced nasal symptoms in this study.^24^^,^^33^

To overcome the problem of non-standardised and non-reproducible pollen exposure during the season in clinical trials, an allergen exposure chamber (AEC) is an existing option. A certified and validated AEC is a highly standardised platform to perform clinical studies with allergic patients and reliably generate allergic symptoms.^34^ Type and amount of pollen as well as duration of exposure, temperature, and humidity are standardised (after validation studies were performed), leading to study results that are reproducible and more comparable than natural exposures, which can vary between pollen seasons.^35^^,^^36^ The clinical efficacy of a probiotic in Japanese cedar pollen allergy was successfully shown in an AEC study.^26^ However, to the best of our knowledge, there have been no prior reports on the use of an AEC in the evaluation of the efficacy of probiotics in the treatment of birch pollen-induced rhinoconjunctivitis.

The current study is the first confirmatory study with patients suffering from ARC due to birch pollen, investigating the effects of the daily intake of a symbiotic food supplement for 4 months by a controlled provocation in an AEC before (baseline) and after (final) intake.

## Methods

### Study design

**Fig. 1:**
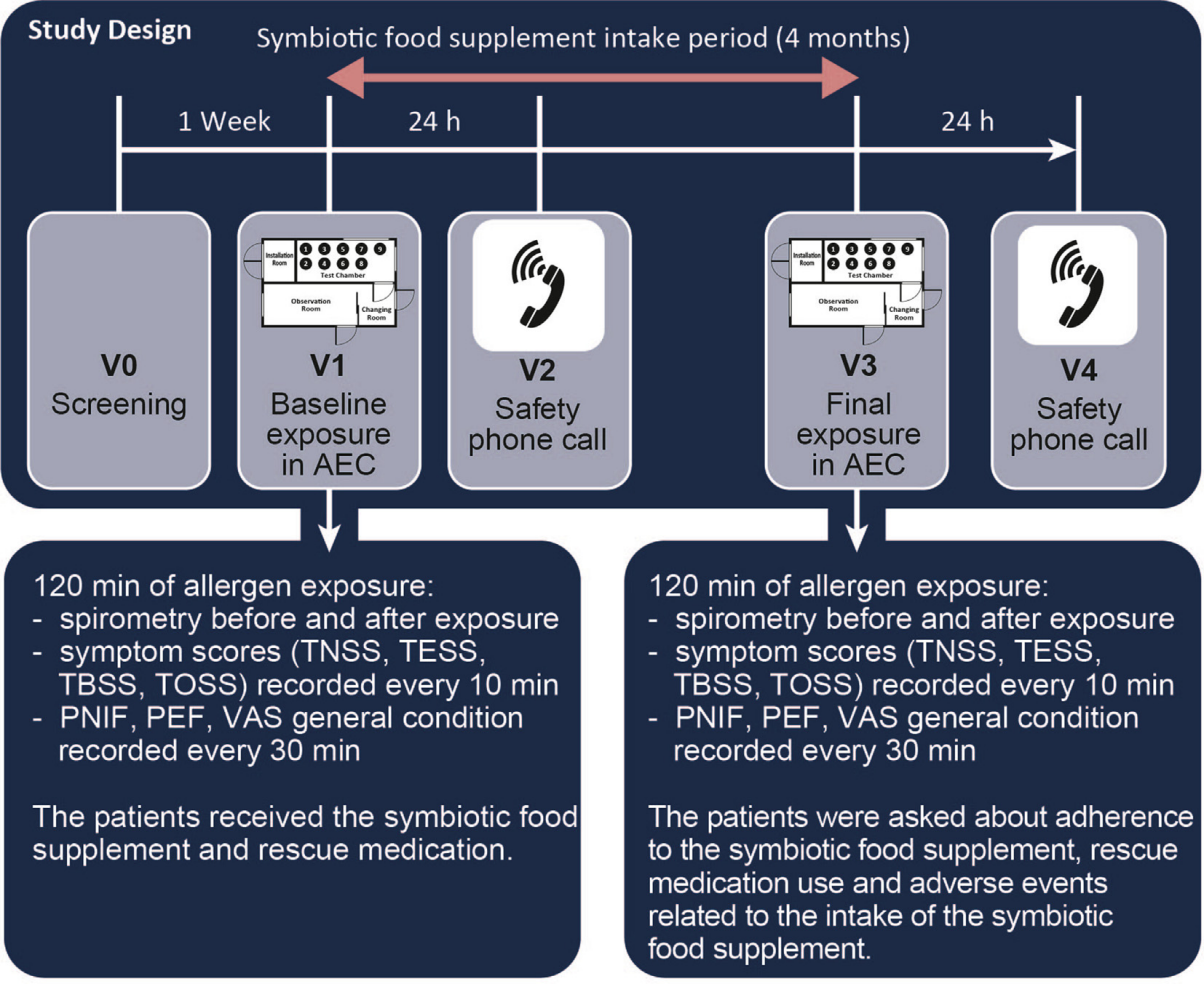
Study design scheme. The scheme shows the visits (V0–V4) during the study period including the parameters recorded at each visit. (V: visit, AEC: Allergen Exposure Chamber, TNSS: Total Nasal Symptom Score, TESS: Total Eye Symptom Score, TBSS: Total Bronchial Symptom Score, TOSS: Total Other Symptom Score, PNIF: Peak Nasal Inspiratory Flow, PEF: Peak Expiratory Flow, VAS: Visual Analogue Scale)

The prospective study “257-P-19, Probiotics_Birch17” investigated the effect of a symbiotic food supplement in patients with rhinoconjunctivitis symptoms induced by birch pollen (Fig. 1). Between June 2019 and January 2020, patients who met the eligibility criteria at screening (visit 0, V0) were exposed to birch pollen at the GA^2^LEN AEC of ECARF Institute Berlin (Germany) at baseline (V1) as well as at the final visit (V3), both outside the birch pollen season. A safety call (V2 and V4) was performed 24 h after both exposures.

Between V1 and V3 all patients were provided with the symbiotic food supplement consisting of a combination of the probiotic strains *Lactobacillus acidophilus NCFM* and *Bifidobacterium lactis BL-04*, as well as the prebiotic Fructo-Oligosaccharides (FOS). The patients took 1 sachet per day as suggested by the instructions for use, for 4 months.^24^^,^^32^

### Patient population

Patients were eligible if they were aged 18–65 years, non-smokers or had quit smoking at least 1 year ago, with a documented history of clinically relevant birch pollen sensitization (skin prick test [SPT] wheal diameter to birch ≥ 3 mm), and rhinoconjunctivitis symptoms induced by birch pollen for at least 2 years according to the ARIA criteria. All patients had to have a positive nasal provocation test (NPT) to birch pollen and/or a reaction to birch pollen in an AEC of TNSS ≥ 3 before this study.

The main exclusion criteria were allergen immunotherapy (AIT) to birch pollen during the last 5 years, clinically relevant hypersensitivity to the ingredients of the symbiotic product, clinically relevant sensitization to grass pollen, house dust mite (*Dermatophagoides pteronyssinus, Dermatophagoides farinae*) and/or cat allergen, severe or uncontrolled asthma during the 3 months before screening, FEV_1_ < 80% predicted before allergen exposure, and relevant infectious or severe chronic diseases or contraindication to adrenaline and/or other rescue medication.

### Allergen Exposure Chamber

The GA^2^LEN AEC of ECARF Institute is a mobile flexible chamber of two standard 7.32 m High-Cube-Containers that can be connected to a unit of 7.45 m × 4.90 m x 2.86 m.^34^^,^^35^^,^^36^ In the standardised and validated chamber, exposure is performed with 8000 natural, non defatted *Betula pendula* pollen/m³ (white birch, *betula verrucosa*) for 120 min at 21 °C and 55% relative air moisture, after an acclimatisation period without exposure for 20 min. During a validation study in this AEC with different exposure periods and doses of birch pollen, the 8000 pollen/m³ for 120 min were shown to best reflect the known symptoms during the season and the Total Nasal Symptom Score (TNSS) being the best clinical parameter.^35^^,^^36^

### Outcomes

During the exposure, Total Nasal Symptom Score (TNSS: sum of the 4 nasal symptoms runny nose, sneezing, itchy nose, and blocked nose), Total Eye Symptom Score (TESS: sum of the 4 eye symptoms itchy eyes, redness, watery eyes, and gritty feeling), Total Bronchial Symptom Score (TBSS: sum of the 4 bronchial symptoms wheezing, cough, breathlessness, and asthma) and Total Other Symptom Score (TOSS: sum of the 2 symptoms itchy palate, and itchy skin) were evaluated every 10 min by the patients. Each single symptom was rated on a scale of 0–3 (no symptoms, mild symptoms, moderate symptoms, and severe symptoms). The Total Symptom Score (TSS) is the sum of TNSS, TESS, TBSS, and TOSS, leading to a maximum of 42.

Co-primary endpoints were the change in TNSS and TSS after 120 min exposure to birch pollen in the AEC at V1 compared to V3. Further exploratory endpoints were the temporal evolution during 120 min exposure and the difference between those temporal trends between baseline (V1) and end of study (V3) in TNSS, TESS, TBSS, TOSS and TSS; changes in Visual Analogue Scale (VAS, personal well-being from 0 = very good to 10 = very bad), Peak Nasal Inspiratory Flow (PNIF, Peak Nasal Inspiratory Flow Meter, Clement Clarke International Ltd., Harlow, Essex, UK) and Peak Expiratory Flow (PEF, Peak-Flow-Meter, Personal Best, Philips GmbH, Herrsching, Germany) recorded at 0, 30, 60, 90, 120 min of exposure, spirometry (FEV_1_, FEV_1_/FVC, MEF_25-75_, EasyOne™ Spirometer, ndd Medizintechnik AG, Zürich Schweiz) performed before and after exposure. Adverse events (AEs) related to the symbiotic product were recorded during the entire study and a safety call performed 24 h after each exposure in the chamber to collect late phase reactions or AEs related to the exposure.

### Study oversight

The study protocol was approved by the Ethics Committee of the Charité, Berlin (EA1/098/17). All participants received detailed information from the study doctor and gave their written informed consent to participate and agreed that their data will be processed and saved according to the General Data Protection Regulation. The study was conducted in accordance with the Declaration of Helsinki and in compliance with all federal, local, or regional requirements. The study was sponsored by Bencard Allergie GmbH. All data provided are pseudonymised to respect the privacy of patients who have participated in the study in line with applicable laws and regulations.

### Statistical analysis

The study was planned as a confirmatory study with 24 patients including some exploratory endpoints. The two co-primary endpoints were analysed using paired Wilcoxon-Test (Wilcoxon signed rank test with continuity correction) and the corresponding p-values were adjusted for multiple testing using the Bonferroni correction.

Percent changes between visits were calculated by first calculating the median of values measured in V1 and V3 separately over all patients. The following equation was applied to obtain percent changes: [(median V3 – median V1)/median V1]∗100. Median and interquartile ranges (IQR) are given together with percent changes.

Symptom scores, VAS, PNIF, and PEF were analysed for their linear evolution over time using linear mixed effects models with patients as random effects accounting for inter-individual variability in baseline symptom scores and treatment (before vs after intake of the symbiotic product), time, and interaction between treatment and time as fixed effects. Model assumptions were checked visually with quantile-quantile plots. Analyses were performed with R version 3.5.3^37^ using package “lme4” for mixed effects modelling^38^ and package “multcomp” for estimating p-values of fixed effects.^39^ Apart from 95% confidence intervals for fixed effects in linear mixed effects models, p-values are given as descriptive summary measures not as results of confirmatory testing. For comparison, mean symptom scores over all patients for all 13 measurements were calculated and presented with 95% confidence intervals. The changes of PNIF and PEF were judged from the point of clinical relevance and described using median and inter quartile ranges.

## Results

### Baseline demographics and disease characteristics

Thirty rhinoconjunctivitis patients with or without asthma fulfilling the inclusion criteria started the study and were exposed at V1. 27 patients (11 with, 16 without asthma) completed the study and were included in the data analysis. Mean age was 45 years (SD 11.8 years), and 15% were male (Table 1). All included patients rated at least 2 symptoms (runny nose, blocked nose, itchy nose, sneezing, itchy eyes) as moderate or severe before study start. The three drop-outs were not related to the intake of the symbiotic food supplement.

**Table 1:** Demographic and baseline Characteristics

Age (years)	N = 27	Mean: 44.8 (SD^b^: 11.8)	Median: 45.00	Min^c^: 23.00	Max^d^: 65.00

Gender n (%)	N=27	Male: 4 (15)		Female: 23 (85)	
Smoker n (%)	N=27	Yes: 0 (0)		No: 27 (100)	
AIT^a^ > 5 years n (%)	N=27	Yes: 10 (37)		No: 17 (63)	

a Allergen Immunotherapy

b Standard deviation

c Minimum

d Maximum

### Efficacy

**Fig. 2:**
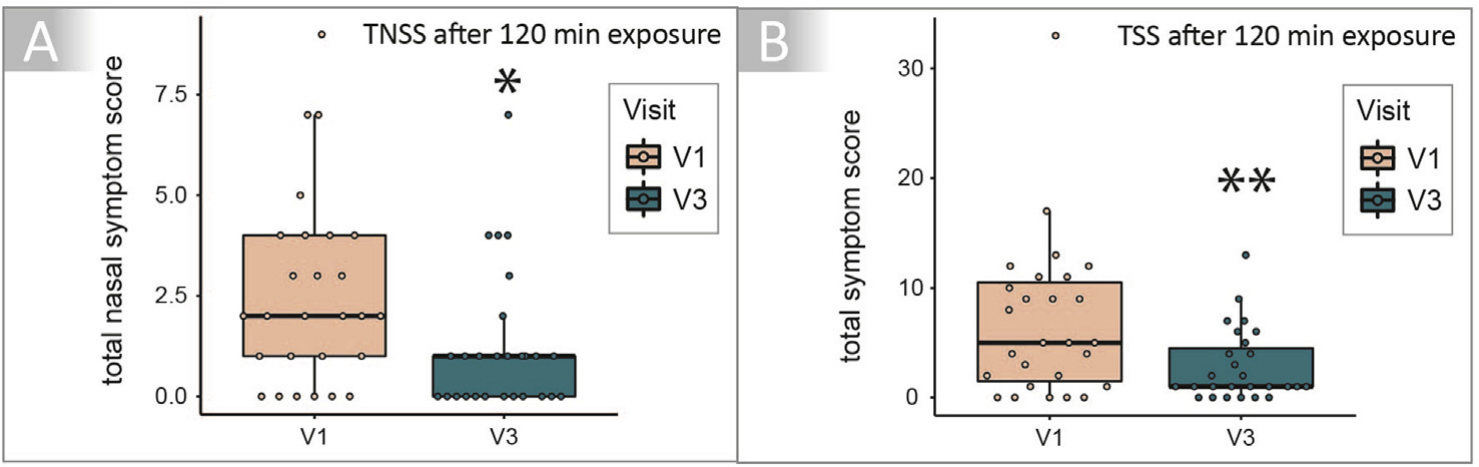
Co-primary endpoints Total Nasal Symptom Score (TNSS) and Total Symptom Score (TSS) at baseline and after 4 months of symbiotic intake evaluated at 120 min birch pollen exposure. The co-primary endpoints A: Total Nasal Symptom Score (TNSS) and B: Total Symptom Score (TSS) showed a significant improvement at V3 (blue) after the symbiotic intake period compared to V1 (red) analysed at 120 min in the AEC. Median TNSS at V1 after 120 min was 2 (IQR: 1–4) and 1 (IQR: 0–1) at V3, describing a significant difference of −50% (adjusted p-value = 0.025). The median TSS at 120 min exposure in the AEC was significantly reduced from 5 (IQR: 1.5–10.5) at V1 to 1 (IQR: 1–4.5) at V3 after treatment, marking a clinically relevant reduction of 80% (adjusted p-value = 0.0097). (∗p-value <0.05, ∗∗p-value <0.01)

At both exposures (V1 and V3), patients recorded their nasal (TNSS), eye (TESS), bronchial (TBSS),and other (TOSS) symptoms every 10 min over a period of 120 min. Co-primary endpoints of the study were the differences between V3 and V1 for TNSS and TSS at 120 min exposure (Fig. 2). The median TNSS at V1 after 120 min was 2 (IQR: 1–4) compared to 1 (IQR: 0–1) at V3, which describes a significant difference of −50% (adjusted p-value = 0.025). In addition, also the median TSS at 120 min exposure in the AEC was significantly reduced from 5 (IQR: 1.5–10.5) at V1 to 1 (IQR: 1–4.5) at V3 after treatment with the symbiotic food supplement, which marks a clinically relevant reduction of 80% (adjusted p-value = 0.0097).

**Fig. 3:**
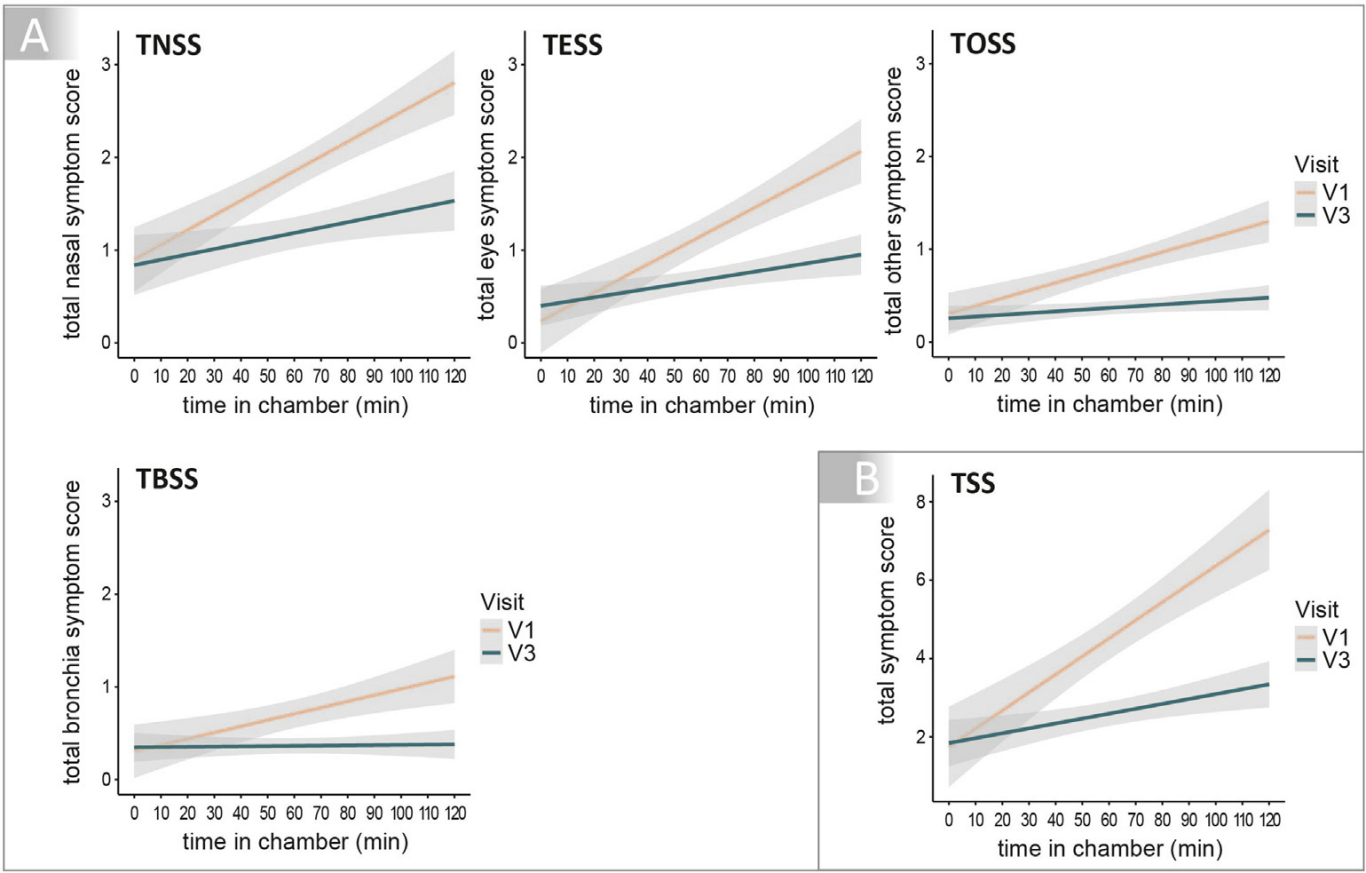
Symptom scores (Total Nasal Symptom Score (TNSS), Total Eye Symptom Score (TESS), Total Bronchial Symptom Score (TBSS), Total Other Symptom Score (TOSS), Total Symptom Score (TSS)) analysed for their linear evolution over time of birch pollen exposure in the AEC at baseline and after 4 months of symbiotic intake. A: TNSS, TESS, TBSS and TOSS were recorded every 10 min during the 120 min exposure and the sum of the four scores is depicted as TSS (B). All four scores decreased to a relevant extent during the 120 min of birch pollen exposure in the Allergen Exposure Chamber (AEC) after the treatment period (V3) compared to baseline (V1)

For all 4 symptom scores, we observed a remarkable improvement of symptoms visible by a weaker increase in symptoms over time (Fig. 3A). The linear mixed effects models, which adjusted for inter-individual variability by estimating a random intercept per patient, identified relevant fixed effects for time in the AEC and relevant interaction effects between time spent in AEC and treatment with the symbiotic product for all 4 symptom scores. The results for all scores are summarised in Table 2. During 120 min of exposure to birch pollen in the AEC the TNSS increased by 0.016 per minute (95% CI: 0.012–0.020, p < 10^−15^) on average during V1. After 4 months of intake of the symbiotic, the slope was decreased by 0.010 per minute (95% CI: 0.015 to 0.0046 decrease, p = 0.00030) identified by the interaction term in the linear mixed effects model. Mean TNSS was reduced from 1.85 (95% CI: 1.66–2.04) during V1 to 1.16 (95% CI: 1.01–1.36) during V3, corresponding to a relevant reduction of 0.66 points (−36%) (differences between values are based on non-rounded numbers, which also applies for the subsequent calculations).

**Table 2:** Results for all scores

	V1 mean score (95% CI)	V3 mean score (95% CI)	Difference	V1 slope/min (95% CI)	p	Slope decrease between V1 and V3/min (95% CI)	p	V3 slope/min
TNSS	1.85 (1.66–2.04)	1.16 (1.01–1.36)	- 36%	0.016 (0.012–0.020)	<10^−15^	0.010 (0.015–0.0046)	0.0003	0.006
TESS	1.15 (0.96–1.34)	0.68 (0.56–0.79)	- 41%	0.015 (0.012–0.019)	<10^−16^	0.011 (0.015–0.006)	<10^−5^	0.004
TBSS	0.71 (0.56–0.86)	0.36 (0.28–0.45)	- 49%	0.0067 (0.0044–0.0091)	<10^−7^	0.0065 (0.0098–0.0031)	0.0002	0.0002
TOSS	0.80 (0.68–0.93)	0.37 (0.30–0.44)	- 54%	0.0083 (0.0064–0.010)	<10^−15^	0.0064 (0.0091–0.0038)	<10^−5^	0.0019
TSS	4.5 (3.9–5.1)	2.6 (2.3–2.9)	- 43%	0.046 (0.037–0.055)	<10^−15^	0.034 (0.047–0.021)	<10^−6^	0.012

This table summarizes the results of the linear mixed effects model, analysing the symptom scores for their linear evolution over time. The mean scores (95% CI) during V1 and V3 and the percentage difference between baseline (V1) and final (V3) exposure are given. The slope per minute (95% CI) is given for V1 and the slope decrease between V1 and V3 per minute (95% CI) is shown including p-values as descriptive summary measures. V3 slope per minute is the difference between (V1 slope/min – slope decrease between V1 and V3/min)

Results for the TESS were comparable, the TESS increased by 0.015 per minute (95% CI: 0.012–0.019, p < 10^−16^) on average during V1. After treatment, the slope was decreased. The mean TESS was reduced from 1.15 (95% CI: 0.96–1.34) during V1 to 0.68 (95% CI: 0.56–0.79) during V3, describing a relevant reduction of 0.48 points (−41%).

During V1 the TBSS increased by 0.0067 on average per minute (95% CI: 0.0044–0.0091, p < 10^−7^). After the symbiotic intake period, the slope was decreased. Mean TBSS was reduced from 0.71 (95% CI: 0.56–0.86) during V1 to 0.36 (95% CI: 0.28–0.45) during V3, showing a relevant reduction of 0.34 points (−49%).

Itching palate is an often reported symptom in allergic rhinoconjunctivitis. The mean TOSS was reduced from 0.80 (95% CI: 0.68–0.93) during V1 to 0.37 (95% CI: 0.30–0.44) during V3, corresponding to a relevant reduction of 0.44 points (−54%).

Fig. 3B shows the development of the TSS, the sum of symptoms of the nose, eyes, bronchial system, and others for each time point during 120 min provocation with birch pollen in the AEC. The TSS increased by 0.046 per minute (95% CI: 0.037–0.055, p < 10^−15^) on average during V1. After treatment, the change over time was decreased. Mean TSS was reduced from 4.5 (95% CI: 3.9–5.1) during V1 to 2.6 (95% CI: 2.3–2.9) during V3. This corresponds to a clinically relevant reduction of 1.9 points (−43%) for the sum of all symptoms for the total population.

**Fig. 4:**
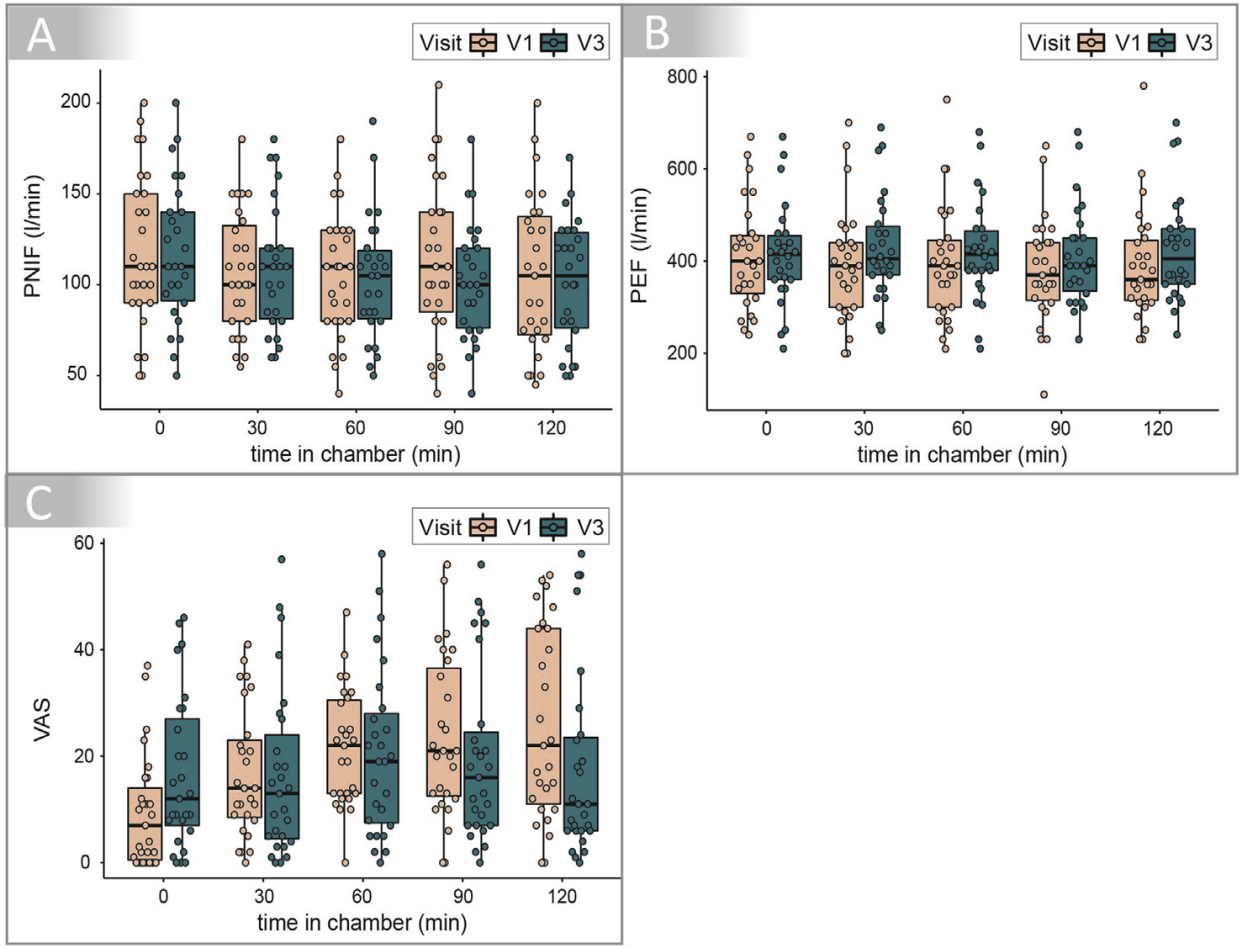
Peak Nasal Inspiratory Flow (PNIF), Peak Expiratory Flow (PEF) and Visual Analogue Scale (VAS) representing the personal well-being during 120 min of birch pollen exposure in the AEC at baseline and after 4 months of symbiotic intake. A: PNIF (l/min), B: PEF (l/min) and C: personal well-being (VAS) were recorded every 30 min during the 120 min exposure. PNIF and PEF did not show a relevant difference after the treatment period (V3) compared to baseline (V1). The personal well-being recorded via VAS showed a relevant improvement after the treatment period (V3) compared to baseline (V1)

There were no relevant differences observed for the Peak Nasal Inspiratory Flow (PNIF) (Fig. 4A). Also spirometry parameters (not shown) did not exhibit relevant differences between V1 and V3, however, since there were no obstructions or restrictions measured before and after exposure at baseline, differences were not expected at V3.

The Peak Expiratory Flow (PEF) decreased slightly after 60 min of exposure in the AEC at V1 from median PEF 390 L/min (IQR: 300–445) to median PEF 370 L/min after 90 min (IQR: 315–440) and median PEF 360 after 120 min (IQR: 315–445) (Fig. 4B). This decrease was not clinically relevant. After 4 months of symbiotic intake at V3, the PEF values remained stable during exposure (median ranged from 390 to 415 for the five measurements) and showed a slight tendency for an improved value at 120 min exposure since PEF was 405 after 120 min (IQR: 350–470) compared to 360 during V1. The personal well-being of the patients, evaluated via Visual Analogue Scale (VAS, lower values represent better well-being) was improved at the second exposure (V3) (Fig. 4C). At the end of the exposure after 120 min median VAS was reduced by 50% between V1 (median = 22, IQR: 11–44) and V3 (median = 11, IQR: 6–23.5), which represents a relevant increase of the patient's personal well-being.

In the safety calls 24 h after baseline exposure with birch pollen (V2), 11 patients reported late phase reactions due to the exposure: itchy and tearing eyes, irritated or sore throat, thirst, swallowing problems, dyspnoea, obstructed nose, headache, itchy skin and mild urticaria, cough, and sneezing. During the safety call 24 h after end of the second exposure (V4) only 3 patients reported late phase reactions: sneezing, mild cough, chest tightness, or mild dyspnoea, indicating an increase in tolerability to the pollen exposure after the intake of the symbiotic food supplement.

### Safety

No AEs related to the food supplement were reported during the intake period and the safety calls.

## Discussion

The protective farm effect on the development of diseases like allergy and asthma has been intensively discussed for many years and efforts have been taken to transform these findings into therapeutic concepts. The consumption of unpasteurised (raw) cow's milk seems to be an important part of the farm effect by contributing to the low prevalence of asthma and allergies in farm children.^40^ However, the consumption of raw milk cannot be recommended due to the risk of life-threatening infections. Interestingly, there are experimental concepts to profit from the benefits of raw cow's milk, but bypassing the risks coming with its consumption. An idea, which is currently being evaluated in a clinical trial, is the consumption of minimally processed cow's milk, which is microbiologically safe, but still has heat-labile molecules and more components in the fat fraction compared to industrially processed milk.^40^ Roth-Walter et al. recently published another approach in this direction, a study on the protective effect of loaded beta-lactoglobulin, the major whey protein in cow's milk, in prevention and therapy of allergy.^41^ The authors showed that the treatment with iron-quercetin-loaded beta-lactoglobulin protected mice from sensitization and had the ability to impair mast cell degranulation. Beta-lactoglobulin was detected in the air of cow sheds, implying a potential route for mucosal tolerance induction via the nasal epithelium and therefore probably delivering an important part of the mechanism of the protective farm effect.^42^ Additionally, beneficial bacterial strains or components, isolated from stables, were shown to induce protection and likely contribute to the farm effect.^2^^,^^3^ Probiotics with proven beneficial bacterial strains (eg, also combined with prebiotics) are therefore a reasonable option in allergy prevention and therapy. Nasal probiotic products have been studied and a recent study showed that the beneficial *Lactobacillus casei AMBR2* can rapidly colonize the nasal epithelium and withstand nasal clearance to a certain extent. This study was performed only in healthy subjects and the beneficial properties should be confirmed in patients.^43^ The usefulness of oral pro- and symbiotic products as an adjunct therapy in allergic diseases was discussed controversially in the past and solid clinical data was requested.^44^^,^^45^

In this study, the effects of an orally administered symbiotic food supplement, taken daily for 4 months, were investigated in birch pollen allergic patients for the very first time in a highly standardised and validated allergen exposure chamber before and after intake of the symbiotic product. Patients observe comparable symptoms as during the allergy season, when exposed to birch pollen in the AEC. This procedure can be reproduced in the same setting, with the same method and the same amount of pollen after the intake period, enabling the patient to realise improvement or worsening of the symptoms. Patients record their symptoms during the exposure period of 120 min, the cumulative effect of pollen exposure can be realised and symptoms of different organs like eyes, nose, and respiratory tract can be collected separately. This increases the reliability of the results.^35^ Although sample size was not calculated during the planning phase for this first evaluation of a symbiotic food supplement in birch pollen allergy in an AEC, we defined the number of participating patients as being adequate and feasible to investigate the objectives of the study. Nevertheless, the lack of pre-study sample size estimation is a limitation of our study.

The probiotic/prebiotic combination led to robust and clinically relevant treatment effects after an intake period of only 4 months in adults with rhinoconjunctivitis with or without asthma induced by birch pollen, which was shown by the significant reduction in Total Nasal Symptom Score (TNSS) and Total Symptom Score (TSS) analysed after 120 min of birch pollen exposure in the AEC. Nasal, conjunctival, bronchial and other symptoms were decreased to a clinically relevant extent during the final exposure. The late phase reactions induced by the AEC provocation procedure were markedly reduced at the end of the study as a marker for better tolerability of the birch allergen and the personal well-being of the patients increased at the final visit compared to baseline. In addition, the symbiotic food supplement showed an excellent safety and tolerability profile.

The only other study evaluating a probiotic (*Bifidobacterium longum BB536*) in the field of allergic rhinitis in an AEC was done in subjects allergic to Japanese Cedar Pollen (JCP).^26^ The intake of a probiotic twice daily for 4 weeks compared with placebo in 21 patients was investigated in a double-blind, placebo-controlled, crossover study. The study had no baseline AEC exposure, the exposure after end of treatment with a comparable setting (6500–7000 pollen/m^3^, 4 h exposure period, symptoms recording every 30 min) indicated that compared to placebo, the probiotic intake was associated with significant less ocular symptoms, starting after 80 min. No improvement in nasal, throat or disruption of normal activities during pollen exposures in patients with JCP allergy were shown. During the days after JCP exposure an improvement of both, the scores for the disruption of normal activities and the need for medication use, could be observed. In our study the intake of the symbiotic product with two different strains for 4 months resulted in much higher efficacy shown as significant reduction in TNSS and TSS. The late phase reactions, inflammatory reactions that occur several hours after contact with the allergen contributing to cell damage, characterized by tissue-infiltrating eosinophils and T lymphocytes, were also considerably reduced after treatment.

A limitation of our study is the missing placebo control. Placebo effects play a fundamental role when estimating magnitudes of efficacy of treatment options in all diseases.^46^ In the field of allergen immunotherapy, several factors impact this effect.^46^ It has to be kept in mind that subjects in a placebo-group of an AIT trial are allowed to use symptomatic rescue medication, which relieve symptoms to a certain extent. Therefore, medication scores are recorded in addition to symptom scores in these trials. A previously reported placebo effect in a double blind placebo controlled (DBPC) trial with subcutaneous immunotherapy in birch pollen allergic patients was 6%–18% with comparable allergen exposure during the seasons based on the area under the curve (AUC) of the symptom medication score during season.^47^ The placebo effect in DBPC trials using AECs ranged from +11% to +0,5% after 6 months using the TNSS (−7% after 4 months in 1 trial), and −4% to −17% after 6 months using the Total Ocular Symptom Score in house dust mite (HDM) allergic patients with sublingual immunotherapy (SLIT), and was −18.5% in grass SLIT (Rhinoconjunctivitis Total Symptom Score after 4 months) and −18.8% in birch subcutaneous immunotherapy (SCIT) (TSS after 10 weeks).^48^ The efficacy of the symbiotic product in our study exceeded these previously reported effects of placebo groups in AEC trials with a reduction in the median TNSS of 50% and median TSS of 80%. So, even without being able to control for placebo effects in this study, we are confident that the symbiotic product shows clinically relevant improvement for patients with allergic rhinitis. This efficacy shown in a robust clinical setting and the excellent safety and tolerability profile qualifies this symbiotic product as an adjuvant approach in the management of allergic rhinoconjunctivitis with or without asthma. The identification of new and novel strains might further improve this new therapeutic approach in the near future.

## Conclusions

The paradigm of the human microbiome, hygiene hypothesis, and microbial diversity and its relationship to dysbiosis and distinct diseases is a fascinating concept attracting increasing attention. This first evaluation of a symbiotic food supplement in an allergen exposure chamber in birch pollen allergic patients, suffering from rhinoconjunctivitis with or without asthma, under standardised conditions revealed a significant beneficial effect, harnessing significant improvements of symptoms and well-being while maintaining an excellent safety and tolerability profile. This confirmatory study adds valuable information, which is sought by the community, about the effectiveness of a specific symbiotic product as an adjuvant approach in the management of allergic rhinoconjunctivitis and is the cornerstone for further controlled clinical studies.

## Funding

This study was funded by Bencard Allergie GmbH, Leopoldstr. 175, 80,804 München.

## Data availability

The datasets generated during and/or analysed during the current study are not publicly available. Bencard Allergie GmbH is committed to sharing with qualified external researchers, access to patient-level data, and supporting clinical documents from related studies. These requests are reviewed and approved by an independent review panel on the basis of scientific merit.

## Author contributions

KCB, JH, TM, MFK and SG designed the study. Patient enrollment and follow-up were done by KCB, SB and SK. Study conduction and study quality control were done by KCB and TZ. Data acquisition was performed by KCB, SB and SK. LK performed the statistical analysis. KCB, OP, SG and AG evaluated and interpreted the data. LK and AG prepared the manuscript together with a medical writing service. All authors reviewed and approved the final manuscript version and its submission.

## Ethics approval and consent to participate

The study protocol was approved by the Ethics Committee of the participating centre (Charité Berlin, Germany; EA1/098/17). The study was conducted in accordance with the Declaration of Helsinki and in compliance with all federal, local, or regional requirements. Informed consent was obtained from all participants. All data provided are pseudonymised to respect the privacy of patients who have participated in the study in line with applicable laws and regulations.

## Consent for publication

The authors provide their consent for the publication of the study results.

## Declaration of competing interest

JH, MT, MFK, SG, and AG are employees of Allergy Therapeutics / Bencard Allergie GmbH. Ms. Becker, Dr. Krause and Mr. Kugler have nothing to disclose. Prof. Bergmann reports personal fees for Lectures: ALK, AstraZeneca, Allergopharma, Almirall, Bencard, Chiesi, GSK, HAL, LETI, Lofarma, Mundipharma, Novartis, Sanofi. Non-financial support as Chair of German Pollen Information Service Foundation, personal fees and non-financial support from Consultant physician for ECARF, personal fees and non-financial support from Advisory Board member of AstraZeneca, ECARF, GSK, Robert-Koch-Institute Berlin (Vice chairman Public Health), Sanofi, outside the submitted work. Prof. Pfaar reports grants and personal fees from ALK-Abelló, grants and personal fees from Allergopharma, grants and personal fees from Stallergenes Greer, grants and personal fees from HAL Allergy Holding B.V./HAL Allergie GmbH, grants and personal fees from Bencard Allergie GmbH/Allergy Therapeutics, grants and personal fees from Lofarma, grants from Biomay, grants from Circassia, grants and personal fees from ASIT Biotech Tools S.A., grants and personal fees from Laboratorios LETI/LETI Pharma, personal fees from MEDA Pharma/MYLAN, grants and personal fees from Anergis S.A., personal fees from Mobile Chamber Experts (a GA2LEN Partner), personal fees from Indoor Biotechnologies, grants from Glaxo Smith Kline, personal fees from Astellas Pharma Global, personal fees from EUFOREA, personal fees from ROXALL, personal fees from NOVARTIS, personal fees from SANOFI AVENTIS, personal fees from Med Update Europe GmbH, personal fees from streamedup! GmbH, outside the submitted work. Prof. Zuberbier reports personal fees from Bayer Health Care, FAES, Novartis, Henkel, and AstraZeneca. He received fees for talks and personal fees from AbbVie, ALK, Almirall, Astellas, Bayer Health Care Fee, Bencard Allergie GmbH, Berlin Chemie, HAL, Leti, Meda, Menarini, Merck, MSD, Novartis, Pfizer, Sanofi, Stallergenes, Takeda, Teva, UCB Henkel, Kryolan, L'Oréal outside the submitted work. The other authors declare that there are no competing interests.

## References

[1] Agache I, Akdis CA, Chivato T, Hellings P, Hoffmann-Sommergruber K, Jutel M. EAACI white paper on research, innovation and quality Care. http://www.eaaci.org 2018. Accessed 15 Jun 2020.

[2] von Mutius E., Braun-Fahrlander C., Schierl R. (2000). Exposure to endotoxin or other bacterial components might protect against the development of atopy. Clin Exp Allergy.

[3] Debarry J., Garn H., Hanuszkiewicz A. (2007). Acinetobacter lwoffii and Lactococcus lactis strains isolated from farm cowsheds possess strong allergy-protective properties. J Allergy Clin Immunol.

[4] Cahenzli J., Koller Y., Wyss M., Geuking M.B., McCoy K.D. (2013). Intestinal microbial diversity during early-life colonization shapes long-term IgE levels. Cell Host Microbe.

[5] Kummeling I., Stelma F.F., Dagnelie P.C. (2007). Early life exposure to antibiotics and the subsequent development of eczema, wheeze, and allergic sensitization in the first 2 years of life: the KOALA Birth Cohort Study. Pediatrics.

[6] Irani C., Hallit S., Mouzannar M., Salameh P. (2019). Aeroallergen sensitization and upper respiratory allergies among patients living in rural and urban areas: real-life exploration of the hygiene hypothesis. World Allergy Organization Journal.

[7] Matricardi P.M. (2010). 99th Dahlem conference on infection, inflammation and chronic inflammatory disorders: controversial aspects of the 'hygiene hypothesis'. Clin Exp Immunol.

[8] von Hertzen L., Hanski I., Haahtela T. (2011). Natural immunity Biodiversity loss and inflammatory diseases are two global megatrends that might be related. EMBO Rep.

[9] Hufnagl K., Pali-Scholl I., Roth-Walter F., Jensen-Jarolim E. (2020). Dysbiosis of the gut and lung microbiome has a role in asthma. Semin Immunopathol.

[10] Mennini M., Fierro V., Di Nardo G., Pecora V., Fiocchi A. (2020). Microbiota in non-IgE-mediated food allergy. Curr Opin Allergy Clin Immunol.

[11] FAO/WHO (2006). Probiotics in Food. Health and Nutritional Properties and Guidelines for Evaluation FAO Food and Nutritional.

[12] Hill C., Guarner F., Reid G. (2014). The International Scientific Association for Probiotics and Prebiotics consensus statement on the scope and appropriate use of the term probiotic. Nat Rev Gastroenterol Hepatol.

[13] Kramer M.F., Heath M.D. (2014). Probiotics in the treatment of chronic rhinoconjunctivitis and chronic rhinosinusitis. J Allergy.

[14] Fiocchi A., Pawankar R., Cuello-Garcia C. (2015). World allergy organization-McMaster university guidelines for allergic disease prevention (GLAD-P): probiotics. World Allergy Organ J.

[15] Cuello-Garcia C.A., Fiocchi A., Pawankar R. (2016). World allergy organization-McMaster university guidelines for allergic disease prevention (GLAD-P): prebiotics. World Allergy Organ J.

[16] Pfefferle P.I., Prescott S.L., Kopp M. (2013). Microbial influence on tolerance and opportunities for intervention with prebiotics/probiotics and bacterial lysates. J Allergy Clin Immunol.

[17] Sharma G., Im S.H. (2018). Probiotics as a potential immunomodulating pharmabiotics in allergic diseases: current status and future prospects. Allergy Asthma Immunol Res.

[18] Klimek L., Schmidt-Weber C.B., Kramer M.F., Skinner M.A., Heath M.D. (2017). Clinical use of adjuvants in allergen-immunotherapy. Expet Rev Clin Immunol.

[19] Fox A., Bird J.A., Fiocchi A. (2019). The potential for pre-, pro- and synbiotics in the management of infants at risk of cow's milk allergy or with cow's milk allergy: an exploration of the rationale, available evidence and remaining questions. World Allergy Organization Journal.

[20] Tan-Lim C.S., Esteban-Ipac N.A. (2018). Probiotics as treatment for food allergies among pediatric patients: a meta-analysis. World Allergy Organization Journal.

[21] Hsiao K.C., Ponsonby A.L., Axelrad C., Pitkin S., Tang M.L. (2017). Long-term clinical and immunological effects of probiotic and peanut oral immunotherapy after treatment cessation: 4-year follow-up of a randomised, double-blind, placebo-controlled trial. Lancet Child Adolesc Health.

[22] Perrin Y., Nutten S., Audran R. (2014). Comparison of two oral probiotic preparations in a randomized crossover trial highlights a potentially beneficial effect of Lactobacillus paracasei NCC2461 in patients with allergic rhinitis. Clin Transl Allergy.

[23] Wassenberg J., Nutten S., Audran R. (2011). Effect of Lactobacillus paracasei ST11 on a nasal provocation test with grass pollen in allergic rhinitis. Clin Exp Allergy.

[24] Ouwehand A.C., Nermes M., Collado M.C., Rautonen N., Salminen S., Isolauri E. (2009). Specific probiotics alleviate allergic rhinitis during the birch pollen season. World J Gastroenterol.

[25] Xiao J.Z., Kondo S., Yanagisawa N. (2006). Effect of probiotic Bifidobacterium longum BB536 [corrected] in relieving clinical symptoms and modulating plasma cytokine levels of Japanese cedar pollinosis during the pollen season. A randomized double-blind, placebo-controlled trial. J Investig Allergol Clin Immunol.

[26] Xiao J.Z., Kondo S., Yanagisawa N. (2007). Clinical efficacy of probiotic Bifidobacterium longum for the treatment of symptoms of Japanese cedar pollen allergy in subjects evaluated in an environmental exposure unit. Allergol Int.

[27] Wang M.F., Lin H.C., Wang Y.Y., Hsu C.H. (2004). Treatment of perennial allergic rhinitis with lactic acid bacteria. Pediatr Allergy Immunol.

[28] Lin T.Y., Chen C.J., Chen L.K., Wen S.H., Jan R.H. (2013). Effect of probiotics on allergic rhinitis in Df, Dp or dust-sensitive children: a randomized double blind controlled trial. Indian Pediatr.

[29] Lin W.Y., Fu L.S., Lin H.K., Shen C.Y., Chen Y.J. (2014). Evaluation of the effect of Lactobacillus paracasei (HF.A00232) in children (6-13 years old) with perennial allergic rhinitis: a 12-week, double-blind, randomized, placebo-controlled study. Pediatr Neonatol.

[30] Ishida Y., Nakamura F., Kanzato H. (2005). Clinical effects of Lactobacillus acidophilus strain L-92 on perennial allergic rhinitis: a double-blind, placebo-controlled study. J Dairy Sci.

[31] Singh A., Hacini-Rachinel F., Gosoniu M.L. (2013). Immune-modulatory effect of probiotic Bifidobacterium lactis NCC2818 in individuals suffering from seasonal allergic rhinitis to grass pollen: an exploratory, randomized, placebo-controlled clinical trial. Eur J Clin Nutr.

[32] Manzotti G., Heffler E., Fassio F., obotSS Group (2014). Multi-strain symbiotic preparations as a novel adjuvant approach to allergic rhinitis. Columbia International Publishing Journal of Contemporary Immunology.

[33] Gelardi M., De Luca C., Taliente S. (2017). Adjuvant treatment with a symbiotic in patients with inflammatory non-allergic rhinitis. J Biol Regul Homeost Agents.

[34] Pfaar O., Calderon M.A., Andrews C.P. (2017). Allergen exposure chambers: harmonizing current concepts and projecting the needs for the future - an EAACI Position Paper. Allergy.

[35] Zuberbier T., Abelson M.B., Akdis C.A. (2017). Validation of the Global Allergy and Asthma European Network (GA(2)LEN) chamber for trials in allergy: innovation of a mobile allergen exposure chamber. J Allergy Clin Immunol.

[36] Voegler T., Goergen F., Bergmann K.C. (2017). Technical specification of the Global Allergy and Asthma European Network (GA2LEN) chamber: a novel mobile allergen exposure chamber Allergo. Journal International.

[37] R Core Team (2018). R: A Language and Environment for Statistical Computing. https://www.R-project.org/.

[38] Bates D., Maechler M., Bolker B., Walker S. (2015). Fitting Linear Mixed-Effects Models Using Lme4. Journal of Statistical Software.

[39] Hothorn T., Bretz F., Westfall P. (2008). Simultaneous inference in general parametric models. Biom J.

[40] Brick T., Hettinga K., Kirchner B., Pfaffl M.W., Ege M.J. (2019). The beneficial effect of farm milk consumption on asthma, allergies, and infections: from meta-analysis of evidence to clinical trial. JACI: In Pract.

[41] Roth-Walter F., Pacios L.F., Gomez-Casado C. (2014). The major cow milk allergen Bos d 5 manipulates T-helper cells depending on its load with siderophore-bound iron. PLoS One.

[42] Pali-Schöll I., Bianchini R., Hofstetter G. (2020). Bauernhofeffekt anders gedacht: bovines Beta-Laktoglobulin kommt zusammen mit seinen Liganden im Stallstaub vor und zeigt in vitro einen Allergie-protektiven Effekt. Allergo J Int.

[43] De Boeck I., van den Broek M.F.L., Allonsius C.N. (2020). Lactobacilli have a niche in the human nose. Cell Rep.

[44] Ege M.J., Frei R., Bieli C. (2007). Not all farming environments protect against the development of asthma and wheeze in children. J Allergy Clin Immunol.

[45] Perkin M.R., Strachan D.P. (2006). Which aspects of the farming lifestyle explain the inverse association with childhood allergy?. J Allergy Clin Immunol.

[46] Frew A.J., Pfaar O. (2018). Placebo effects in allergen immunotherapy: an experts' opinion. Allergo J Int.

[47] Narkus A., Lehnigk U., Haefner D., Klinger R., Pfaar O., Worm M. (2013). The placebo effect in allergen-specific immunotherapy trials. Clin Transl Allergy.

[48] Pfaar O., Agache I., Bergmann K.C. (2020 Apr 23). Placebo Effects in Allergen Immunotherapy - an EAACI Task Force Position Paper. Allergy.

